# Integrating international policy standards in the implementation of postnatal care: a rapid review

**DOI:** 10.1136/bmjgh-2023-014033

**Published:** 2023-01-24

**Authors:** Helen Smith, Aleena M Wojcieszek, Shuchita Gupta, Antonella Lavelanet, Åsa Nihlén, Anayda Portela, Marta Schaaf, Marcus Stahlhofer, Özge Tunçalp, Mercedes Bonet

**Affiliations:** 1 International Health Consulting Services Ltd, Liverpool, UK; 2 Department of Sexual and Reproductive Health and Research including UNDP/UNFPA/UNICEF/WHO/World Bank Special Programme of Research, Development and Research Training in Human Reproduction (HRP), World Health Organization, Geneva, Switzerland; 3 Mater Research Institute, The University of Queensland, Brisbane, Queensland, Australia; 4 Department of Maternal, Newborn, Child and Adolescent and Health and Ageing, World Health Organization, Geneva, Switzerland; 5 Independent Consultant, Brooklyn, New York, USA

**Keywords:** Health policy, Maternal health, Health systems, Public Health, Review

## Abstract

**Introduction:**

International legal and political documents can assist policy-makers and programme managers in countries to create an enabling environment to promote maternal and newborn health. This review aimed to map and summarise international legal and political documents relevant to the implementation of the WHO recommendations on maternal and newborn care for a positive postnatal experience.

**Methods:**

Rapid review of relevant international legal and political documents, including legal and political commitments (declarations, resolutions and treaties) and interpretations (general comments, recommendations from United Nations human rights treaty bodies, joint United Nations statements). Documents were mapped to the domains presented in the WHO postnatal care (PNC) recommendations; relating to maternal care, newborn care, and health systems and health promotion interventions, and by type of human right implied and/or stated in the documents.

**Results:**

Twenty-nine documents describing international legal and political commitments and interpretations were mapped, out of 45 documents captured. These 29 documents, published or entered into force between 1944 and 2020, contained content relevant to most of the domains of the PNC recommendations, most prominently the domains of breastfeeding and health systems interventions and service delivery arrangements. The most frequently mapped human rights were the right to health and the right to social security.

**Conclusion:**

Existing international legal and political documents can inform and encourage policy and programme development at the country level, to create an enabling environment during the postnatal period and thereby support the provision and uptake of PNC and improve health outcomes for women, newborns, children and families. Governments and civil society organisations should be aware of these documents to support efforts to protect and promote maternal and newborn health.

**WHAT IS ALREADY KNOWN ON THIS TOPIC**

- The transformative power of postnatal care (PNC) has yet to be fully realised due to suboptimal uptake and quality of the care provided. International legal and political documents, including those relating to human rights, can promote an enabling environment to facilitate the implementation of maternal and child health programmes by tying such efforts to the essential human rights that states are obliged to respect, protect and fulfil.

**WHAT THIS STUDY ADDS**

- To our knowledge, this is the first time international legal and political documents have been reviewed and summarised specifically to determine their relevance to the postnatal period and to the implementation of PNC. It shows that existing documents contain content that is relevant to most of the domains of the WHO recommendations on maternal and newborn care for a positive postnatal experience, most often in the areas of breastfeeding and health systems interventions and service delivery arrangements.

**HOW THIS STUDY MIGHT AFFECT RESEARCH, PRACTICE OR POLICY**

- Legislators, policy-makers, programme managers and advocates can leverage the synergies between the WHO PNC recommendations and human rights principles to create an enabling environment for PNC at the country level and maximise the immediate and longer-term health and well-being of women, newborns and their families after childbirth.

## Introduction

The postnatal period is defined as the period immediately following the birth of the newborn to 6 weeks (42 days) after birth.^1^ It is a period where postpartum women and newborns face high burden of mortality and morbidity.^2^ A crucial component of the maternal and newborn care continuum, postnatal care (PNC) services provide the platform for the care of women and newborns during this critical period. PNC includes care of the woman and the newborn, as well as health systems policies and programmes designed to support families and improve the quality of PNC. The ultimate objective of PNC is to prevent complications after childbirth, promote healthy practices, garner support from broader families and communities, and meet health, developmental and social needs.^2^ Yet the importance of PNC has long been overlooked by policymakers, health workers and communities^3–5^ due to complex, multifactorial issues, including potentially diminished perceived urgency and importance of PNC relative to antenatal and intrapartum care. This has resulted in poor coverage and quality of care for women and newborns,^6^ and lost opportunities to promote health and well-being, particularly among disadvantaged groups.^7^

In 2022, the WHO published the WHO recommendations on maternal and newborn care for a positive postnatal experience.^2^ This updated and expanded guideline reflects an important global shift in the exclusive focus of care; from reducing death and serious illness following birth to comprehensive, holistic care and support to promote health and well-being; to ensuring that women and newborns survive and thrive. It describes a ‘positive postnatal experience’ as the desired endpoint for all women, partners, parents, caregivers and families after birth. A positive postnatal experience is defined as one in which women and families receive information and reassurance in a consistent manner from motivated health workers, and where both the women’s and babies’ health, social and developmental needs are recognised, within a resourced and flexible health system that respects their cultural context.^2^

International legal and political documents are key tools to advance global health, human rights and equity in sexual and reproductive health.^8 9^ These documents can help to reduce health inequalities, influence social and structural determinants of health, support stronger health systems, and create healthier and safer workplaces and communities.^10 11^ To inform policy dialogue and integrated planning of programmes, WHO has created a compendium of health systems policies that support delivery of reproductive, maternal, newborn and child health interventions across multiple sectors.^9^ The WHO Maternal, Newborn, Child and Adolescent Health and Ageing Data Portal contains legal and policy data, including national policies, strategies, laws and regulations.^12 13^ These data allow for analysis of the policy environment and its effects on maternal and newborn health.^14 15^ There is also a global abortion policies database^16^ that includes all relevant legislation, guidelines and constitutions to promote accountability and transparency with regard to national abortion laws and practices. Further, in the area of respectful maternity care, WHO has mapped pathways between types of mistreatment and their connection with human rights standards, and explored how human rights treaty bodies have expressed these issues to inform strategies within health systems.^17 18^

A variety of international documents exist to support women, newborns and families in the postnatal period, which can facilitate an enabling environment for the implementation of the PNC guideline. Yet, in many countries, insufficient legislative commitments have been made for priority indicators on maternity protection (measures to protect the health of the woman and newborn and provide employment and income security during maternity) or regulation against the marketing of breastmilk substitutes.^19 20^ There is no comprehensive mapping of international legal and political documents (such as treaties, conventions, and interpretations of legal and political commitments) relevant to the postnatal period and the implementation of PNC. Providing a mapping of how these documents relate to the WHO recommendations on PNC could thereby help national legislators, policymakers, programme managers, and civil society organisations strengthen the design and/or review of existing policies, strategies and practices to create an enabling and empowering environment for PNC. Such a mapping should also help to position PNC within international standards and human rights-based frameworks, through which states are obliged to respect, protect and fulfil human rights as per the commitments they have made under international human rights law.^21 22^

This rapid review aimed to identify the available international legal and political documents relevant to the postnatal period and PNC, and determine how the content of these documents pertains to the implementation of the WHO recommendations on maternal and newborn care for a positive postnatal experience.^2^

## Methods

This review followed established approaches to rapid review,^23 24^ where systematic review methods related to topic refinement, setting eligibility criteria, searching, study selection, data extraction and other review components are streamlined and processes accelerated to complete the review efficiently in a short time.^23^

### Inclusion and exclusion criteria

International documents comprising legal and political commitments (declarations, resolutions and treaties), interpretations (general comments, recommendations from United Nations (UN) human rights treaty bodies, joint UN statements) and other non-binding documents (action plans, strategies, frameworks, resolutions, political declarations) pertinent to the postnatal period and PNC were included. Documents about specific population groups or documents that were not expressly relevant to the postnatal period or PNC were excluded. Table 1 lists the inclusion and exclusion criteria.

**Table 1:** Inclusion and exclusion criteria

Criterion	Included	Excluded
Population	Women, newborns, mothers, fathers, parents, family members	Documents about specific population groups (eg, women living with HIV, persons living with disabilities)
Intervention/scope	Declarations, treaties, conventions, policies or strategies that mention maternity care, including postnatal care	Documents that do not mention keywords related to the postnatal period (eg, postnatal, postpartum, breast feeding, newborns).
Context	Global documents	Documents produced at the regional or subnational level
Outcomes	Motherhood/self-care, family and newborn care practices, or women’s/parents’ responsibilities	Broader rights-based outcomes (eg, safety, environment, economic, development)
	Workplace/work or employment conditions/maternity or paternity or family benefits	
	Right to health and support services (eg, any of the right to health elements)	
	Breastfeeding maintenance/protection from harmful marketing	
Type of document	Documents produced as part of the UN system (eg, UN funds, programmes, specialised agencies, entities and bodies and related organisations)	Documents produced by advocacy or special interest groups or professional associations
	High-level global documents including treaties, conventions, resolutions, policies, strategies	Documents such as annual reports, reports of research or studies, discussion papers, evidence or policy briefs, position statements, guidelines (likely to need updating)
Language	Global documents published in English	Non-English language documents (if documents were available in other languages, the English language version was used)
Date limits	No date limits (conventions are sometimes adopted and re-adopted from the original; in which case we planned to use the latest version)	_

UN, United Nations.

### Search strategy

Relevant documents were identified via (1) general internet searches, (2) websites of relevant organisations and (3) direct contact with informants, as below.

1. General internet search: General Google and Google Scholar searches were designed to locate relevant global documents, along with literature referring to conventions, laws and policies relevant to PNC and the rights of women, newborns and family. Searches were carried out using keywords relating to conventions, policies and strategies, PNC and human rights, social protection and employment policies (online supplemental table S1).
2. Websites of relevant organisations: Search functions within the websites of specific organisations were used to identify relevant internal documents (online supplemental table S2). Simple one-word or two-word combinations were used, or else advanced searching was used where available.
3. Direct contact with key informants: Direct email contact was made with individuals working in relevant UN agencies, or other academic centres or groups known to be working in the area and able to locate relevant documents (eg, Centre for Human Rights and International Justice, Stanford University; Carr Centre for Human Rights Policy, Harvard University; Geneva Centre for Human Rights Advancement and Global Dialogue). Where documents were sourced internally, or through colleagues or key informants within specific agencies/organisations/departments, no additional internet or website searches were carried out for those agencies/organisations/departments (see online supplemental table S3).

10.1136/bmjgh-2023-014033.supp1 Supplementary data

Correspondence with key informants and initial web searches (phase 1 of the search) were carried out in May 2021 and June 2021, respectively. Key informants were contacted again in January 2023 (phase 2 of the search) to check whether any additional documents had been published prior to submission of the current manuscript.

### Screening and selection

A database of all documents retrieved was created and continually updated by HS, with support from AMW. One author (HS or AMW) screened the titles and abstracts/summary text of all records and a second author (MB) independently verified 25% of the records. Full-text documents were assessed against the inclusion criteria by one author (HS or AMW) and a second author (MB) independently verified 25%. Results were compared and discrepancies resolved by discussion and returning to the documents. Reasons for exclusion of any documents were recorded.

### Data extraction

Key data from all included documents were extracted using a predefined data extraction pro forma in Microsoft Excel. The data extraction pro forma was piloted on 2–3 documents to ensure fit for purpose and relevancy of defined fields. Data extraction fields included type of document, year of publication, individual or organisation responsible for developing the document, summary of main purpose, and a categorisation of the type of right relevant to the implementation of the PNC recommendations. Fields included in the data extraction pro forma and examples of data extracted are presented in online supplemental table S4.

### Assessment of risk of bias

The purpose of the review was to provide an overview of the available international legal and political documents; critical appraisal of the documents including risk of bias was not required.

### Categorisation of documents for mapping against WHO PNC domains

Eligible documents were categorised into (1) those not applicable for the mapping against the WHO PNC domains and (2) those applicable for the mapping:

1. Documents not applicable for the mapping against WHO PNC domains: Key international legal documents are pieces of international human rights law that signatory countries are obliged to implement and provide frameworks from which legal and political commitments and interpretations of these commitments have been developed. While highly relevant, these documents were not applicable for the mapping as they do not provide the granularity needed for PNC implementation as opposed to the subsequent, more specific documents derived from them. Broad statements on human rights and health, or women’s, children’s and adolescent’s health that outline the application of human rights approaches to health and the realisation of the health rights of women, children and adolescents were also deemed inapplicable for the mapping, as these were considered less relevant for the implementation of programmes or recommendations. For example, many of these documents are reports of UN Special Rapporteurs, with a mandate to monitor specific human rights or rights-relevant issues or raise the profile of specific issues pertinent to the protection of human rights, many of which relate to health. The result of these reports, and other strategies or roadmaps produced by UN agencies, may ultimately support improved uptake of PNC services.
2. Documents applicable for mapping against WHO PNC domains: These included international legal and political commitments and interpretations of these commitments. International legal and political commitments include political declarations and resolutions to act, while interpretations of legal and political commitments often serve to clarify country reporting duties and suggest approaches for implementation. These documents provide important standard-setting and guidance and are oriented towards action relevant to the implementation of PNC.

### Synthesis of findings

Included documents were summarised and categorised based on the data extracted in the pro forma. A mapping of documents against the PNC domains, as presented in the WHO PNC guideline, was produced in a table . The domains relate to (postpartum) maternal care (maternal assessment, interventions for common physiological signs/symptoms, preventive measures, maternal mental health interventions, nutritional interventions and physical activity, and postpartum contraception); newborn care (newborn assessment, preventive measures, nutrition interventions, infant growth and development, and breast feeding); and health systems and health promotion interventions (social, behavioural and community interventions and health system interventions and service delivery arrangements). The specific types of interventions addressed by the guideline within each PNC domain are listed in online supplemental table S5).

The documents for inclusion in the mapping were then scrutinised to determine the type of right, as stated or implied in the document, namely the (1) right to health (including the right to the highest attainable standard of health; equal access to care and a health system enabling environment for breastfeeding);(2) right to social security (including paid leave; maternity protection; family benefits and breastfeeding arrangements at work);(3) right to information (related to protection from harmful marketing) and (4) other human rights relevant to PNC (including the right to identity (legal registration of the birth); freedom from torture and other ill treatment; and right to decide the number, spacing and timing of children).

### Patient and public involvement

Patients and the public were not directly involved in this review. All sourced documents were publicly available. Results will be shared via social media, and with key stakeholders involved in the adaptation and implementation of the WHO PNC recommendations.

The authors of this manuscript recognise gender diversity among birthing individuals. In line with the WHO recommendations on maternal and newborn care for a positive postnatal experience, this manuscript uses the terms ‘woman’or ‘mother’as inclusive of all individuals who have given birth, even if they may not identify as a woman or as a mother.

## Results

### Document flow

**Figure 1:**
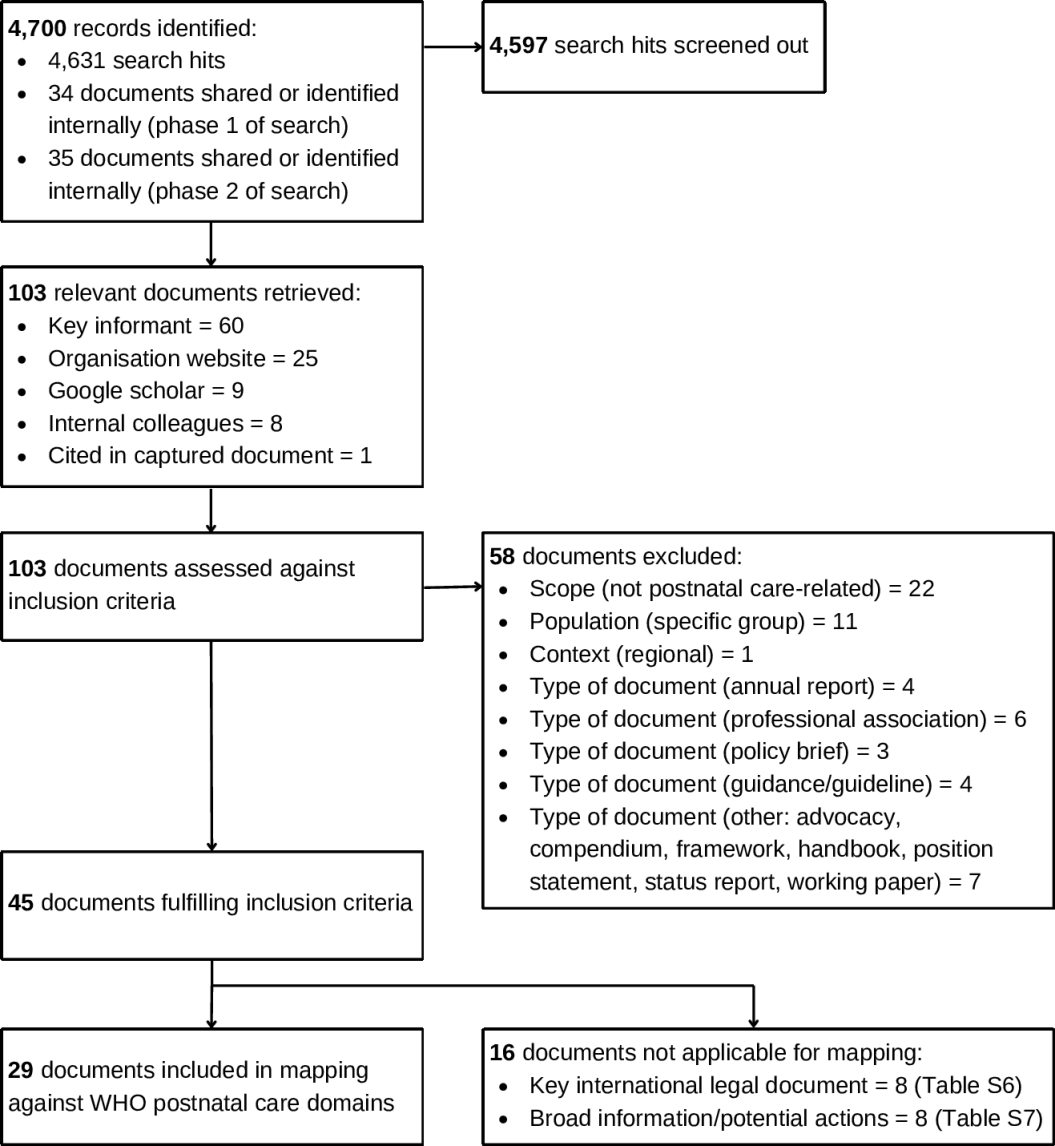
Flow diagram.

Figure 1 presents the document flow. In total, 4700 records were identified from searches, shared by key informants, or sourced internally. After removing duplicates and records deemed irrelevant based on screening of titles or summary text, 103 relevant documents underwent detailed review. Fifty-eight documents were excluded based on ineligible scope, population, context or document type. The remaining 45 eligible documents comprised 16 key international legal documents or broad statements on human rights (not applicable for the mapping exercise) and 29 international legal and political commitments and interpretations of these commitments (described in the following section).

### Documents not applicable for mapping against WHO PNC domains (n=16)

Eight key international legal documents were adopted or entered into force between 1948 and 2000. These documents are listed box 1, with further detail provided in online supplemental table S6). Another eight documents containing broader statements on human rights and health, or women’s, children’s and adolescent’s health were published between 2004 and 2021 and are listed in online supplemental table S7.

Box 1 **Key international legal documents relevant to postnatal care**

1. Universal Declaration of Human Rights.
2. C102—Social Security Convention.
3. International Covenant on Civil and Political Rights.
4. International Covenant on Economic, Social and Cultural Rights.
5. Convention on the Elimination of All Forms of Discrimination against Women.
6. C156—Workers with Family Responsibilities Convention.
7. Convention on the Rights of the Child.
8. C183—Maternity Protection Convention.

See online supplemental table S6 for more details.

### Documents included in mapping against WHO PNC domains (n=29)

**Figure 2:**
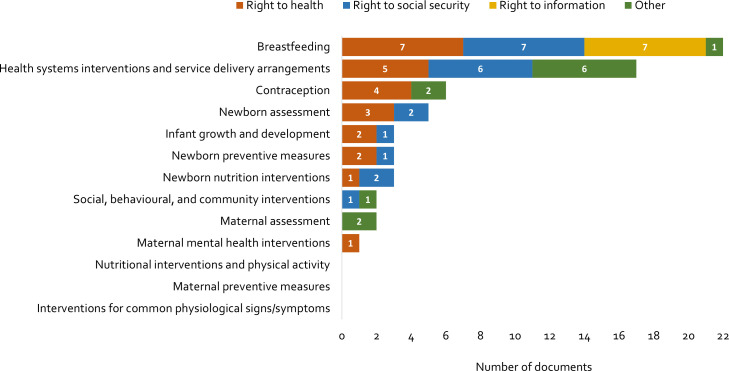
International legal and political documents supporting the implementation of postnatal care by type of right and postnatal care domain.

Table 2 presents the mapping of the 29 documents by PNC domain and type of right (NB: domains in which no documents were identified are not shown in table 2; these related to maternal health in the areas of nutritional interventions and physical activity, preventive measures and interventions for common physiological signs/symptoms). Figure 2 summarises the distribution of documents mapped across the 13 PNC domains, which highlight at a glance where supportive legal and political commitments or interpretations of these commitments exist, and where gaps are evident.

**Table 2:** Mapping of international legal and political commitments and interpretations against postnatal care domains

International legal and political commitments and interpretations of legal and political commitments (n=29)	Postnatal care domains											
	Maternal care*				Newborn care						Health systems and health promotion interventions	
	Maternal assessment	Mental health interventions	Contraception		Newborn assessment	Preventive measures	Nutrition interventions	Infant growth and development	Breastfeeding		Social, behavioural, and community interventions	Health systems interventions and service delivery arrangements
Legal and political commitments (n=12)												
HRC Resolution 22/7: Birth registration and the right of everyone to recognition everywhere as a person before the law2013												†
HRC Resolution 28/13: Birth registration and theright of everyone to recognition everywhere as a person before the law2015												†
HRC Resolution 33/11:Preventable mortality andmorbidity of children under 5yearsof age as a human rights concern 2016					‡							‡
HRC Resolution 33/18: Preventable maternal mortality and morbidity and human rights 2016			‡	§							§	
HRC Resolution adopted by the Human Rights Councilon 24 March 2017.34/15: Birth registration and the right of everyoneto recognition everywhere as a person before the law2017												†
HRC Resolution 41/14: Equal pay 2019									¶		**/††	**/††
HRC Resolution 41/17: Preventing and responding to violence against women and girls in the world of work 2019									§			
HRC Special Rapporteur A/74/137: A human-rights based approach to mistreatment and violence against women in reproductive health services with a focus on childbirth and obstetric violence 2019	§											§
Innocenti Declaration on the protection, promotion and support of breastfeeding 1990									‡‡	§§		
									¶			
WHA 27.43: Infant nutrition and breastfeeding 1974									‡‡	§§		
									¶			
WHA 55.25: Infant and youngchild nutrition *2002*									‡‡	§§		
WHA 69: Maternal, infant and young childnutrition: guidance on ending the inappropriate promotion of foods for infants andyoung children.*2016*									‡‡			
Interpretations of legal and political commitments (n=17)												
ICESCR GC No.14: Theright to the highest attainable standard of health Art. 12 *E/C.12/2000/4;2000*			¶¶									¶¶
ICESCR GC No.19: The right to social security (Art.9)*E/C.12/GC/19;2008*					**/††	**/††	**/††	**/††				***/**
ICESCR GC No.22 on the right to sexual and reproductive health Art.12 of the ICESCR *E/C.12/GC/22;2016*			‡									
CEDAW GC No.24: Article 12 of the ConventionWomen and Health *A/54/38/Rev.1, chap. I;1999*		‡	‡/¶¶									¶¶
ICESCR GC No. 25: Science and economic, social and cultural rights Article 15(1)(b),(2), (3) and (4)of the ICESCR *E/C.12/GC/25; 2020*									‡‡	§§		
CRC GC No.15 on the right of the childto the enjoyment of the highest attainable standrd of healthArt.24 CRC/C/GC/15;2013					‡	‡	‡	‡	‡			¶¶
CRC GC No.7: Implementing childrights in early childhoodCRC/C/GC/7/Rev.1;2005					‡	‡		‡				†
ILO: Income Security Recommendation R067 1944												***/**
ILO: Workers with Family Responsibilities Recommendation R165.1981												**
ILO: Maternity Protection Recommendation R1912000									¶			**/††
ILO: Social Protection Floors Recommendation R202 2012					**		**					**
Joint WHO/UNICEF meeting on Infant and YoungChildFeeding: statement, recommendations, listof participants 1981									§§			
									¶			
Joint statement by the UN Special Rapporteurs: Right to Food, Right to Health, Working Group on Discrimination against Women in law and in practice, and RC in support of increased efforts to promote, support and protect breast-feeding 2016									‡‡			
									¶			
The prevention and elimination of disrespect andabuse during facility-based child birth.WHO, 2014	§											§
Ensuring human rights inthe provision of contraceptive information and services.WHO, 2014			†††									
Global strategy for infantand young childfeedingWHO, 2003									‡‡	§§		
									¶			
Ending hospital detention for non-payment of bills: legal and health financing policy optionsWHO, 2020												¶¶

For a summary of content extracted from the documents to inform this mapping exercise, see online supplemental table S8.

Colour key: blue= right to social security; green=other rights; orange=right to health; yellow=right to information.

*Three domains are not shown: no documents were identified in relation to maternal care in the areas of nutritional interventions and physical activity, preventive measures and interventions for common physiological signs/symptoms.

†Other—right to identity.

‡Right to health—highest attainable standard of health.

§Other—right to freedom from torture and other ill treatment.

¶Right to social security—breastfeeding arrangements at work.

**Right to social security—maternity protection.

††Right to social security—family benefits.

‡‡Right to information—related to protection from harmful marketing.

§§Right to health—health system enabling environment for breast feeding.

¶¶Right to health—equal access to care.

***Right to social security—paid leave.

†††Other—right to decide number, spacing and timing of children.

CEDAW, Convention on the Elimination of All Forms of Discrimination against Women; CRC, Convention on the Rights of the Child; GC, general comment; HRC, UN Human Rights Council; ICESCR, Convention on Economic, Social and Cultural Rights; ILO, International Labour Organization; UN, United Nations; WHA, World Health Assembly.

Together, the 29 captured documents contained content relevant to 10 of the 13 PNC domains and were published between 1944 and 2020. The PNC domains for which there were documents containing supportive declarations, statements or recommendations, include breast feeding (addressed 22 times across 6 legal and political commitments and 6 interpretations of legal and political commitments) and health systems interventions and service delivery arrangements for PNC (addressed 17 times across 6 legal and political commitments and 11 interpretations of legal and political commitments). In relation to health systems and service delivery, identified documents covered issues related to birth registration, parental leave and entitlements, equal access to health services and respectful care. No documents contained statements relevant to maternal nutritional interventions and physical activity, preventive measures for the woman, or interventions for common maternal physiological signs/symptoms. Only one document addressed maternal mental health. For a summary of content extracted from the documents to inform this mapping exercise, see online supplemental table S8.

Of the legal and political commitments (n=12), six documents stipulated actions relevant to breast feeding. These included the Innocenti Declaration on the protection, promotion and support of breast feeding^25^ and World Health Assembly (WHA) Resolutions 27.43,^26^ 55.25^27^ and 69^28^ around infant and young child nutrition. Six legal and political commitments were deemed relevant to the provision of health system interventions and service arrangements, including Human Rights Council (HRC) Resolutions 22/7^29^ and 28/13^30^ around birth registration, and HRC Resolution 33/11^31^ on the prevention of mortality and morbidity in children under 5 years of age. Two legal and political commitments (HRC Resolutions 33/18^32^ and 41/14^33^) contained actions applicable to social, behavioural and community interventions for PNC; both referring to policies and healthcare services that address discrimination and equality.

Of the interpretations of legal and political commitments (n=17), six suggested strategies or approaches for implementing or supporting provisions for breastfeeding. These included general comments on International Covenant on Economic, Social and Cultural Rights (ICESCR) (2020)^34^ and the Convention on the Rights of the Child (CRC) (2013),^35^ the Maternity Protection Recommendation R191 (2000),^36^ as well as a joint UN statement on infant and young child feeding (1981).^37^ Eleven of the interpretations of legal and political commitments suggested or clarified human rights provisions relevant to health systems interventions and service delivery arrangements, including general comments on ICESCR (2000 and 2008),^38 39^ the Convention on the Elimination of All Forms of Discrimination against Women (CEDAW) (1999)^40^ and the CRC (2013),^35^ all of which relate to the right to access to care and the right to paid leave and maternity protection. Four interpretations of legal and political commitments offered recommendations for or approaches to implementing rights relevant to the provision of postpartum contraception, including general comments on the ICESCR (2000 and 2016)^39 41^ and on CEDAW (1999),^40^ which all clarify and affirm access to reproductive health services including contraception as a human right.

In terms of type of right, the right to health was the most frequently addressed in the documents (n=14), particularly as it related to achieving the highest attainable standard of health and creating a health system enabling environment for breastfeeding (both n=6). The right to social security was also frequently addressed (n=11), including in terms of breastfeeding arrangements at work (n=7) and maternity protection (n=6). Seven documents addressed the right to information related to protection from harmful marketing of breastmilk substitutes. Some documents focused on more than one type of right; for example, the HRC Resolution 33/18^32^ focused on both the right to the highest attainable standard of health and the right to freedom from torture and other ill treatment; and HRC Resolution 41/14^33^ focused on the right to social security in relation to paid leave, maternity protection and family benefits.

## Discussion

We identified several key international legal documents and broader statements on human rights and health relevant to the postnatal period and PNC. In addition, a mapping of international legal and political commitments and interpretations of these commitments demonstrated content supportive of most of the domains of the WHO recommendations on maternal and newborn care for a positive postnatal experience.^2^ The domains of breastfeeding and health systems and service delivery interventions for PNC included in the WHO guideline had the most documents associated with them, followed by the domain of postpartum contraception. In relation to health systems and service delivery, mapped documents covered issues related to birth registration, parental leave and entitlements, equal access to health services and respectful care. The most frequently mapped human rights covered were the right to health and the right to social security.

Efforts over the last decades in the areas of breastfeeding are notable and translate in the number of documents covering these topics identified in this review. Regarding breastfeeding, duty bearers can be guided by the seven policy priorities to protect, promote and support breastfeeding set out by the Global Breastfeeding Collective.^42^ Its 2021 scorecard indicates action is needed particularly around implementing the code of marketing of breastmilk substitutes, maternity leave and increasing the numbers of baby-friendly health facilities.^43^ The WHO Nutrition Landscape Information System^44^ can be used to track the legal status of the Code in countries.

Several documents included related to maternity/parental leave and breastfeeding in the workplace, and clearly describe the rights and statutory social support that should be available to women and partners as they transition to parenthood and return to work. Parental leave may lead to better maternal, infant and child health^45^ through higher uptake of immunisation, breastfeeding and PNC services.^46 47^ However, the offer of parental leave and other benefits is often limited due to economic constraints, social and political norms, pre-existing inequalities and other complex factors, particularly in low-income and middle-income countries.^19 20 48^

A range of documents also recognise birth registration, the foundation of legal identity,^21^ as crucial for improving maternal, neonatal and child health outcomes and access to healthcare services.^49 50^ However, coverage of birth registration still falls short in many countries, due to barriers to accessing birth registration services, policy and regulatory challenges, and inadequate or absent civil registration systems.^48^ Country efforts to adopt, adapt, and implement PNC recommendations could support integration of birth registration into health services, before discharge from health facilities after birth or at subsequent postnatal contacts.

Continued efforts around postpartum family planning and contraceptive information and services is vital to protecting sexual and reproductive health rights, particularly where barriers such as cost, access to services, and lack of or insufficient education and counselling are present.^51^ A systematic review found that offering modern contraception as part of PNC, and integration of family planning and immunisation services, including intrafacility referrals between services, may increase use of contraceptives and is likely to reduce both unintended pregnancies and pregnancies that are too closely spaced.^52^

In the current review, no documents were identified in relation to maternal care in areas that could be considered as more clinical or health promotion focused, such as nutritional interventions and physical activity, preventive measures and interventions for common physiological signs/symptoms. This may reflect a potentially diminished focus on women’s physical health and well-being following childbirth, as compared with that of the newborn. Such interventions and services are important for women to access after giving birth to obtain the information and support needed to stay healthy and understand when to seek healthcare. Self-care may facilitate PNC implementation with respect not only to these domains, but also around postpartum contraception, and could potentially be translated into policy action.^11^ The WHO guideline on self-care interventions for health and well-being, 2022 revision^53^ outlines evidence-based recommendations to support individuals, communities and countries to adopt self-care interventions, some of which pertain to PNC and can potentially increase access to care and improve health outcomes. Importantly, such interventions should be used in addition to interaction with the health system, not as a replacement.^54^ Only one document addressed maternal mental health interventions, despite the adverse effects of postpartum depression and anxiety on the health and well-being of women and their newborns, and on mother–child relationships. Duty bearers can be guided by the WHO guide for integration of perinatal mental health in maternal and child health services^55^to increase maternal mental health support seeking and provision of quality, respectful, judgement-free care.

Laws, policies and regulations support the fulfilment of human rights. A variety of documents included in this review relate to the right to health, social security, information and identity, among others. They can assist efforts to strengthen the capacity of countries to integrate a human rights-based approach to policies and programmes, advocate for health-related human rights.^22^ Making these links explicit by updating existing policies, strategies and practices may support and encourage all relevant duty bearers to act to support these rights, strengthen the implementation of PNC, help meet the health and social needs of women, newborns, parents and families^9^ and ensure a positive postnatal experience. A human rights-based situation analysis may also help in efforts to redress inequity. Important policies include universal health coverage (UHC), policies to end hospital detention for non-payment of bills and paid parental leave, alongside investment in community-oriented care including accessible maternity centres and outpatient services.^5 56^

While they do not necessarily guarantee action, international standards form a solid foundation for countries to ensure an enabling environment for implementation of the PNC guideline.^9^ As countries implement PNC recommendations, they could be encouraged to analyse their own current laws and policies related to international standards, and adapt their legislations to advance implementation of international standards.^9^ In addition, PNC services provide a platform for integrated provision of health and social services aligned with legislations on birth registration, breastfeeding protection and support, and postpartum contraception. Once country policies and legislations are updated to meet these standards, it becomes imperative to monitor and evaluate their implementation and effects over time. Mechanisms exist to monitor and report on interdisciplinary priority policies and targets,^12 19 20 44 57^ including Sustainable Development Goals (SDG) targets on health, including contraception and other SDGs related to breastfeeding, birth registration, social protection and maternity benefit coverage, sexual and reproductive rights, and ending discrimination and violence against women.

Several documents identified recognise and protect the health and social needs of women, newborns, parents and families in the continuum of pregnancy, women’s and child health.^9 54^ Others have also looked at international documents that have been used to restrict the exposure of children to unhealthy foods and beverage marketing.^58^ Our results should encourage countries to consider wider effects of their legislation on maternal and child health beyond essential PNC. Such effects may relate to care at work during pregnancy (related to healthy working environments, working times, time off for antenatal care, protection from hazardous or unhealthy work),^59^ care of small and sick babies, and child-care beyond first few weeks after birth. Our results should also inspire the human rights standard setting systems at international and regional levels to draw on WHO normative guidance in their standard setting for health, both in terms of strengthening the evidence base of human rights standards and in developing human rights standards around the PNC domains for which there were fewer supportive documents.

The rapid review methodology lent itself well to the nature of the current review, as the objective was to capture and categorise international documents relevant to the implementation of PNC. This objective did not necessitate systematic review of research databases, rigorous assessment of methodological or reporting bias, or other important components of traditional systematic reviews. Nonetheless, while we are confident that the relevant major documents were captured, some less prominent documents may have been missed. As our search focused on documents related specifically to essential, routine PNC, it is possible that other relevant documents were not captured if they referred only to broader maternity care or were specific to pregnancy, experience of care or care for women and newborns with specific conditions or complications. We also recognise that some international documents address issues related to parental leave and entitlements after perinatal death or for parents of preterm or low birthweight infant. In addition, we elected to exclude regional documents due to difficulties in ensuring coverage across all regions. Results are also limited by the scope of the recommendations in the WHO PNC guideline. For example, the PNC guideline or other WHO guideline do not include specific recommendations related to tobacco or alcohol use or exposure during the postnatal period, when strategies and frameworks exist to support governments reduce its harmful use.^60 61^ Lastly, while we provide a summary of the content for each of the documents, no content analysis was performed on the strength of language of the statements relevant to the postnatal period or PNC.

## Conclusion

This rapid review provides a comprehensive mapping of international legal and political documents relevant to facilitating implementation of the WHO recommendations on maternal and newborn care for a positive postnatal experience.^2^ It shows that such documents do exist and their contents support most of the domains of PNC outlined in the WHO guideline. Taking a human rights-based approach to the implementation of the PNC recommendations helps to encourage all duty bearers to better enable women, newborns and families to realise their right to health in the postnatal period and beyond.

## Data Availability

The data relevant to the review are included in the article or uploaded as supplementary information.

## References

[1] World Health Organization. WHO technical consultation on postpartum and postnatal care. Geneva: World Health Organization, 2010. 26269861

[2] World Health Organization. WHO recommendations on maternal and newborn care for a positive postnatal experience. Geneva: World Health Organization, 2022. 35467813

[3] Finlayson K, Crossland N, Bonet M, et al. What matters to women in the postnatal period: A meta-synthesis of qualitative studies. PLoS One 2020;15:e0231415. 10.1371/journal.pone.0231415 32320424 PMC7176084

[4] Sacks E, Finlayson K, Brizuela V, et al. Factors that influence uptake of routine postnatal care: findings on women’s perspectives from a qualitative evidence synthesis. PLoS One 2022;17:e0270264. 10.1371/journal.pone.0270264 35960752 PMC9374256

[5] Langlois EV, Dey T, Iaia DG, et al. Improving policy, financing and delivery of postnatal care services. Bull World Health Organ 2023;101:2–2A. 10.2471/BLT.22.289440 36593785 PMC9795378

[6] World Health Organization. Protect the promise: 2022 progress report on the every woman every child global strategy for women’s, children’s and adolescents’ health (2016-2030). Geneva: World Health Organization and United Nations Children’s Fund, 2022.

[7] Langlois ÉV, Miszkurka M, Zunzunegui MV, et al. Inequities in postnatal care in Low- and middle-income countries: a systematic review and meta-analysis. Bull World Health Organ 2015;93:259–270G. 10.2471/BLT.14.140996 26229190 PMC4431556

[8] Gruskin S, Zacharias K, Jardell W, et al. Inclusion of human rights in sexual and reproductive health programming: Facilitators and barriers to implementation. Glob Public Health 2021;16:1559–75. 10.1080/17441692.2020.1828986 33019904 PMC8475719

[9] World Health Organization. A policy guide for implementing essential interventions for reproductive, maternal, newborn and child health (RMNCH). Geneva: World Health Organization, 2014.

[10] Gostin LO, Monahan JT, Kaldor J, et al. The legal determinants of health: harnessing the power of law for global health and sustainable development. Lancet 2019;393:1857–910. 10.1016/S0140-6736(19)30233-8 31053306 PMC7159296

[11] Ferguson L, Narasimhan M, Gutierrez J, et al. Law, human rights and gender in practice: an analysis of lessons from implementation of self-care interventions for sexual and reproductive health. Sex Reprod Health Matters 2021;29:2105284. 10.1080/26410397.2022.2105284 35975874 PMC9387312

[12] World Health Organization. Maternal, newborn, child and adolescent health and ageing – data portal. Available: https://platform.who.int/data/maternal-newborn-child-adolescent-ageing [Accessed 26 May 2023].

[13] World Health Organization. Sexual, reproductive, maternal, newborn, child and adolescent health: policy survey, 2018-2019: summary report. Geneva: World Health Organization, 2020.

[14] Creanga AA, Dohlsten MA, Stierman EK, et al. Maternal health policy environment and the relationship with service utilization in Low- and middle-income countries. J Glob Health 2023;13:04025. 10.7189/jogh.13.04025 36892948 PMC9997690

[15] Mary M, Maliqi B, Stierman EK, et al. Assessing the neonatal health policy landscape in Low- and middle-income countries: findings from the 2018 WHO SRMNCAH policy survey. J Glob Health 2023;13. 10.7189/jogh.13.04024 PMC9983710 36867415

[16] World Health Organization. Global abortion policies database. Available: https://abortion-policies.srhr.org/ [Accessed 12 Oct 2022].

[17] Zampas C, Amin A, O’Hanlon L, et al. Operationalizing a human rights-based approach to address mistreatment against women during childbirth. Health Hum Rights 2020;22:251–64. 32669805 PMC7348458

[18] Khosla R, Zampas C, Vogel JP, et al. International human rights and the mistreatment of women during childbirth. Health Hum Rights 2016;18:131–43. 28559681 PMC5394989

[19] Stierman EK, Maliqi B, Mary M, et al. Changes in the health systems and policy environment for maternal and newborn health, 2008-2018: an analysis of data from 78 low-income and middle-income countries. Soc Sci Med 2023;321:115765. 10.1016/j.socscimed.2023.115765 36801755 PMC10024243

[20] World Health Organization. Tracking Progress towards Universal Coverage for Reproductive, Newborn and Child Health: The 2017 Report. Washington, DC: United Nations Children’s Fund and the World Health Organization, 2017.

[21] World Health Organization. Leading the realization of human rights to health and through health: report of the High-Level Working Group on the Health and Human Rights of Women, Children and Adolescents. Geneva: World Health Organization, 2017.

[22] World Health Organization. Office of the United Nations High Commissioner for Human Rights. A human rights-based approach to health. Geneva: World Health Organization, 2008.

[23] Garritty C, Gartlehner G, Nussbaumer-Streit B, et al. Cochrane rapid reviews methods group offers evidence-informed guidance to conduct rapid reviews. J Clin Epidemiol 2021;130:13–22. 10.1016/j.jclinepi.2020.10.007 33068715 PMC7557165

[24] Tricco AC, Langlois EV, Straus SE. Rapid reviews to strengthen health policy and systems: a practical guide. Geneva: World Health Organization, 2017.

[25] United Nations Children’s Fund. Innocenti Declaration on the protection, promotion and support of breatfeeding. Florence: UNICEF Innocenti Research Centre, 1990.

[26] World Health Assembly, 27. Infant nutrition and breast-feeding (WHA27.43). World Health Organization, 1974.

[27] World Health Organization. 55th World Health Assembly. Infant and Young Child Nutrition (WHA55.25). World Health Organization, 2002.

[28] World Health Assembly, 69. Maternal, infant and young child nutrition: guidance on ending the inappropriate promotion of foods for infants and young children: report by the Secretariat. World Health Organization, 2016.

[29] United Nations Human Rights Council. Birth registration and the right of everyone to recognition everywhere as A person before the law: resolution adopted by the Human Rights Council, 9 April (A/HRC/RES/22/7). 2013.

[30] United Nations Human Rights Council. Birth registration and the right of everyone to recognition everywhere as A person before the law: resolution adopted by the Human Rights Council, 7 April (A/HRC/RES/28/13). 2015.

[31] United Nations Human Rights Council. Preventable mortality and morbidity of children under 5 years of age as A human rights concern: resolution adopted by the Human Rights Council on 29 September 2016, 6 October 2016 (A/HRC/RES/33/11). 2016.

[32] United Nations Human Rights Council. Resolution adopted by the Human Rights Council on 30 September 2016. Preventable maternal mortality and morbidity and human rights (A/HRC/RES/33/18). 2016.

[33] United Nations Human Rights Council. Equal pay: resolution adopted by the Human Rights Council on 11 July 2019 (A/HRC/RES/41/14). 2019.

[34] General Comment no.25. Science and economic, social and cultural rights (Article 15(1)(b), (2), (3) and (4) of the International Covenant on Economic, Social and Cultural Rights (E/C.12/GC/25). Geneva: United Nations Committee on Economic, Social and Cultural Rights, 2020.

[35] General Comment no.15. The right of the child to the enjoyment of the highest attainable standard of health (Artcile 24) (CRC/C/GC/15). Geneva: United Nations Committee on the Rights of the Child, 2013.

[36] International Labour Organization. R191 Maternity Protection Recommendation. Geneva: International Labour Organization, 2000.

[37] World Health Organization and United Nations Children’s Fund. Joint WHO/UNICEF Meeting on Infant and Young Child Feeding, Geneva 9-12 October 1979: statement, recommendations, list of participants. Geneva: World Health Organization, 1981.

[38] General Comment no.19. The right to social security (Article 9) (E/C.12/GC/19). Geneva: United Nations Committee on Economic, Social and Cultural Rights, 2008.

[39] General Comment no.14. The right to the highest attainable standard of health (Article 12) (E/C.12/2000/4). Geneva: United Nations Committee on Economic, Social and Cultural Rights, 2000.

[40] General Recommendation no.24. Article 12 of the Convention (Women and Health) (A/54/38/Rev.1). United Nations Committee on the Elimination of Discrimination Against Women, 1999.

[41] General Comment no.22. The right to sexual and reproductive health (Article 12 of the International Covenant on Economic, Social and Cultural Rights) (E/C/12/GC/22). Geneva: United Nations Committee on Economic, Social and Cultural Rights, 2016.

[42] Global Breastfeeding Collective, United Nations Children’s Fund, World Health Organization. Global Breastfeeding collective. Available: https://www.globalbreastfeedingcollective.org [Accessed 11 Nov 2022].

[43] Global Breastfeeding Collective, United Nations Children’s Fund, World Health Organization. Global breastfeeding scorecard 2021. Protecting breastfeeding through bold national actions during the Covid-19 pandemic and beyond. Geneva: United Nations Children’s Fund, World Health Organization, 2021.

[44] World Health Organization. Nutrition landscape information system (Nlis). nutrition and nutrition-related health and development data. Global Nutrition Monitoring Framework. Available: https://www.who.int/data/nutrition/nlis/gnmf [Accessed 1 May 2023].

[45] Van Niel MS, Bhatia R, Riano NS, et al. The impact of paid maternity leave on the mental and physical health of mothers and children: a review of the literature and policy implications. Harv Rev Psychiatry 2020;28:113–26. 10.1097/HRP.0000000000000246 32134836

[46] Nandi A, Jahagirdar D, Dimitris MC, et al. The impact of parental and medical leave policies on socioeconomic and health outcomes in OECD countries: a systematic review of the empirical literature. Milbank Q 2018;96:434–71. 10.1111/1468-0009.12340 30277601 PMC6131347

[47] Heymann J, Sprague AR, Nandi A, et al. Paid parental leave and family wellbeing in the sustainable development era. Public Health Rev 2017;38:21. 10.1186/s40985-017-0067-2 29450093 PMC5810022

[48] UNICEF. Birth Registration for Every Child by 2030: Are we on track?. New York: United Nations Children’s Fund (UNICEF), 2019.

[49] Every Woman Every Child. Global strategy for women’s, children’s and adolescents’ health (2016-2030). New York: Every Woman Every Child, 2015.

[50] International Confederation of Midwives. Position statement: birth registration. The Hague: International Confederation of Midwives, 2014.

[51] World Health Organization. Ensuring human rights in the provision of contraceptive information and services: guidance and recommendations. Geneva: World Health Organization, 2014. 24696891

[52] Nabhan A, Kabra R, Ashraf A, et al. Implementation strategies, Facilitators, and barriers to Scaling up and sustaining post pregnancy family planning, a mixed-methods systematic review. BMC Womens Health 2023;23:379. 10.1186/s12905-023-02735-z 37468942 PMC10357879

[53] World Health Organization. WHO guideline on self-care interventions for health and well-being, 2022 revision. Geneva: World Health Organization, 2022.

[54] Ferguson L, Fried S, Matsaseng T, et al. Human rights and legal dimensions of self care interventions for sexual and reproductive health. BMJ 2019;365:l1941. 10.1136/bmj.l1941 31085551 PMC6511940

[55] World Health Organization. WHO guide for integration of perinatal mental health in maternal and child health services. Geneva: World Health Organization, 2022.

[56] Crear-Perry J, Correa-de-Araujo R, Lewis Johnson T, et al. Social and structural determinants of health inequities in maternal health. Journal of Women’s Health 2021;30:230–5. 10.1089/jwh.2020.8882 PMC8020519 33181043

[57] United Nations High Commissioner for Human Rights. Human rights indicators: a guide for measurement and implementation. New York: United Nations, 2012.

[58] Sing F, Mackay S, Cinà M, et al. The utilisation of legal instruments by United Nations actors to restrict the exposure of children to unhealthy food and beverage marketing: a qualitative content analysis of UN instruments. Global Health 2023;19:45. 10.1186/s12992-023-00939-4 37391743 PMC10314450

[59] International Labour Organization. Care at work: Investing in care leave and services for a more gender equal world of work. Geneva; Switzerland: International Labour Organization, 2022.

[60] Nurturing Care for Early Childhood Development. Tobacco control to improve child health and development: thematic brief. Geneva: World Health Organization, 2021.

[61] World Health Organization. Global strategy to reduce the harmful use of alcohol. Geneva: World Health Organization, 2010. 10.2471/BLT.19.241737 PMC7047030 32132758

