# Supplementary material for: Integrating international policy standards in the implementation of postnatal care: a rapid review
Source: BMJ Glob Health. 2023 Jan 24;8(Suppl 2):e014033. doi: 10.1136/bmjgh-2023-014033 (PMC10846851; doi:10.1136/bmjgh-2023-014033)
Supplement: Supplementary data [file bmjgh-2023-014033supp001.pdf]

Supplementary material

Table S1: Key word combinations for Google and Google Scholar searches

Where possible, searches of individual websites will use combinations of keywords derived from the inclusion criteria. For example:

Search line	Search terms
**#1**	woman OR newborn OR mother OR father OR parent OR family
**#2**	convention OR declaration OR legislation OR policy OR guideline OR recommendation OR strategy
**#3**	postnatal OR postpartum OR post-birth
**#4**	parenthood OR maternity OR paternity or family
**#5**	workplace OR work OR labour OR employment
**#6**	human rights OR women's rights OR child rights OR right to health OR labour rights
**#7**	breastfeeding OR breastmilk OR milk substitute
**#8**	#1 AND #2 AND #3 AND #4 AND #5
**#9**	#1 AND #2 AND #3 AND #6
**#10**	#1 AND #2 AND #3 AND #7

Table S2: Organisations targeted for website and archive searches

Document type	Organisations/bodies
**International conventions or declarations**	▪ Council of Europe<br>▪ International Labour Organization (ILO)<br>▪ Organisation for Economic Cooperation and Development (OECD)<br>▪ United Nations Childrens Fund (UNICEF)<br>▪ United Nations Development Fund for Women (UNIFEM)<br>▪ United Nations Development Programme (UNDP)<br>▪ United Nations Population Fund (UNFPA)<br>▪ United Nations Entity for Gender Equality and the Empowerment of Women (UNWomen)<br>▪ United Nations Office of the High Commissioner on Human Rights (OHCHR)<br>- UN Human Rights Council (UNHRC)<br>- Commission on the Elimination of Discrimination Against Women (CEDAW)<br>- Committee on the Rights of the Child (CRC)<br>▪ World Health Assembly (WHA)
**Global policies, guidelines, or recommendations**	▪ Alliance for Health Policy and Systems Research<br>▪ Geneva Foundation for Medical Education and Research (GFMER)<br>▪ International Guideline Library of the Guidelines International Network (GIN)<br>▪ International Monetary Fund (IMF)

Smith H, *et al. BMJ Glob Health* 2024; 8:e014033. doi: 10.1136/bmjgh-2023-014033

Document type	Organisations/bodies
	▪ International Confederation of Midwives (ICM)<br>▪ International Federation of Gynecology & Obstetrics (FIGO)<br>▪ International Pediatric Association (IPA)<br>▪ National Institute for Health & Care Excellence (NICE)<br>▪ WHO Heath Systems Governance and Financing<br>▪ WHO Maternal, Newborn, Child and Adolescent Health (MNCAH)<br>▪ WHO Sexual and Reproductive Health and Research (RHR)<br>▪ WHO Partnership for Maternal, Newborn and Child health (PMNCH)<br>▪ World Bank (WB)

Table S3: Search log

Organisation/body	Date	Keywords/filter	Hits
**Council of Europe**	21/5/21	Human rights /English/pdf/CoE countries	190
		Child rights /English/pdf/CoE countries	109
		Social protection /English/pdf/CoE countries	134
**International Labour Organization (ILO)**	21/5/21	Maternity protection/ English/ Global/ document	43
		Social protection/ English/ Global/ document	144
		Family benefits/ English/Global/document	51
		Paternity/ English/ Global/ publication	151
		Social protection children/ English/ Global/ document	71
		Breastfeeding/English/ Global/publication	146
**Organisation for Economic Cooperation and Development (OECD)**	21/5/21	Maternity /publication	14
		Social protection /publication	13
		Human rights	12
		Right to health	11
**United Nations Childrens Fund (UNICEF)**	21/5/21	Human rights; Child rights; Social protection/ global	126
**United Nations Development Fund for Women (UNIFEM)**	N/A: documents sourced internally, or through colleagues or key informants		
**United Nations Development Programme (UNDP)**	N/A: documents sourced internally, or through colleagues or key informants		
**United Nations Population Fund (UNFPA)**	21/5/21	Human rights /publication	16
**United Nations Entity for Gender Equality and the Empowerment of Women (UN Women)**	21/5/21	Human rights /publication	101
		Child rights /publication	11
		Social protection /publication	25
**United Nations Office of the High Commissioner on Human Rights (OHCHR)**	N/A: documents sourced internally, or through colleagues or key informants		

Organisation/body	Date	Keywords/filter	Hits
**UN Human Rights Council (UNHRC)**	N/A: documents sourced internally, or through colleagues or key informants		
**Commission on the Elimination of Discrimination Against Women (CEDAW)**	N/A: documents sourced internally, or through colleagues or key informants		
**Committee on the Rights of the Child (CRC)**	N/A: documents sourced internally, or through colleagues or key informants		
**World Health Assembly (WHA)**	N/A: documents sourced internally, or through colleagues or key informants		
**Alliance for Health Policy and Systems Research**	N/A: documents sourced internally, or through colleagues or key informants		
**Geneva Foundation for Medical Education and Research (GFMER)**	21/5/21	Human rights	1340
		Child rights	1300
		Breastfeeding	445
**International Guideline Library of the Guidelines International Network (GIN)**	N/A: documents sourced internally, or through colleagues or key informants		
**International Monetary Fund (IMF)**	21/5/21	Maternity	100
		Social protection	10
		Human rights	3
		Women	65
**International Confederation of Midwives (ICM)**	21/5/21	Browsed available policy and practice documents	
**International Federation of Gynecology & Obstetrics (FIGO)**	21/5/21	Browsed available FIGO Statements on women's health rights, health for all, maternal health, newborn health	
**International Pediatric Association (IPA)**	21/5/21	Browsed available IPA statements	
**National Institute for Health & Care Excellence (NICE)**	N/A: documents sourced internally, or through colleagues or key informants		
**WHO Heath Systems Governance and Financing**	N/A: documents sourced internally, or through colleagues or key informants		
**WHO Maternal, Newborn, Child and Adolescent Health (MNCAH)**	21/5/21	Browsed available publications by topic: rights, child health, breastfeeding, maternal health, postpartum/postnatal, newborn health	
**WHO Sexual and Reproductive Health and Research (RHR)**	N/A: documents sourced internally, or through colleagues or key informants		
**WHO Partnership for Maternal, Newborn and Child health (PMNCH)**	21/5/21	Browsed available publications on sexual and reproductive health and rights	
**World Bank (WB)**	N/A: documents sourced internally, or through colleagues or key informants		
			**Total hits = 4,631**

Table S4: Fields included in data extraction pro-forma and data extracted

Data extraction field	Data extracted
**Source**	Source of document (e.g. key informant name, "Google scholar", organisational website, etc)
**Title**	Document title (e.g. "International Covenant on Civil and Political Rights")
**URL**	URL (where applicable)
**Acronym**	Acronym for document title (e.g. "ICCPR")
**Year of publication**	Year cited in document
**United Nations agency/publishing organisation**	Specific organisation or department (e.g. "Office of the High Commissioner for Human Rights")
**Type of document**	"Covenant", "convention", "resolution", etc
**Type of right**	Type of right addressed (e.g. right to health, social protection, employment rights)
**Relevant section, article, or paragraph number**	Part, section, paragraph numbers as indicated (e.g. "Section III. Part A. Para.33")
**Summary of relevant section, article, or paragraph**	An approximately one paragraph summary with key messages highlighted
**Assessment against inclusion criteria**	Include/exclude
**Reason for exclusion**	Where applicable, the reason for exclusion of documents according to predefined criteria. For example, if the type of document was an annual report, position statement, policy brief, or other lower-level document not within the scope of the review
**Included docs: relevant to which PNC recommendation**	Recommendation(s) or principle of the PNC guideline to which the content was relevant (e.g. breastfeeding, maternal clinical assessments, time of discharge, improving utilisation of PNC, access to postnatal care)
**Notes**	Any pertinent notes for reference among the review team

PNC: postnatal care

Table S5. Interventions described within the postnatal care guideline domains

DOMAINS	INTERVENTIONS
MATERNAL CARE	
**Maternal assessment**	Physiological assessment of the woman
	HIV catch-up testing
	Screening for tuberculosis disease
**Interventions for common physiological signs and symptoms**	Local cooling for perineal pain relief
	Oral analgesia for perineal pain relief
	Pharmacological relief of pain due to uterine cramping/involution
	Postnatal pelvic floor muscle training for pelvic floor strengthening
	Non-pharmacological interventions to treat postpartum breast engorgement
	Pharmacological interventions to treat postpartum breast engorgement
**Preventive measures**	Non-pharmacological interventions to prevent postpartum mastitis
	Pharmacological interventions to prevent postpartum mastitis
	Prevention of postpartum constipation
	Prevention of maternal peripartum infection after uncomplicated vaginal birth
	Preventive anthelminthic treatment
	Preventive schistosomiasis treatment
	Oral pre-exposure prophylaxis for HIV prevention
**Mental health interventions**	Screening for postpartum depression and anxiety
	Prevention of postpartum depression and anxiety
**Nutritional interventions and physical activity**	Postpartum oral iron and folate supplementation
	Postpartum vitamin A supplementation
	Physical activity and sedentary behaviour
**Contraception**	Postpartum contraception
NEWBORN CARE	
**Newborn assessment**	Assessment of the newborn for danger signs
	Universal screening for abnormalities of the eye
	Universal screening for hearing impairment
	Universal screening for neonatal hyperbilirubinaemia
**Preventive measures**	Timing of first bath to prevent hypothermia and its sequelae
	Use of emollients for the prevention of skin conditions
	Application of chlorhexidine to the umbilical cord stump for the prevention of neonatal infection
	Sleeping position for the prevention of sudden infant death syndrome
	Immunization for the prevention of infections

Smith H, *et al. BMJ Glob Health* 2024; 8:e014033. doi: 10.1136/bmjgh-2023-014033

DOMAINS	INTERVENTIONS
**Nutrition interventions**	Neonatal vitamin A supplementation
	Vitamin D supplementation for breastfed, term infants
**Infant growth and development**	Whole-body massage
	Early childhood development
**Breastfeeding**	Exclusive breastfeeding
	Protecting, promoting and supporting breastfeeding in facilities providing maternity and newborn services
HEALTH SYSTEMS AND HEALTH PROMOTION INTERVENTIONS	
**Health systems interventions and service delivery arrangements**	Schedules for postnatal care contacts
	Length of stay in health facilities after birth
	Criteria to be assessed prior to discharge from the health facility after birth
	Approaches to strengthen preparation for discharge from the health facility to home after birth
	Midwifery continuity of care
	Task sharing components of postnatal care delivery
	Recruitment and retention of staff in rural and remote areas
	Digital targeted client communication
	Digital birth notifications
**Social, behavioural, and community interventions**	Home visits for postnatal care contacts
	Involvement of men in postnatal care and maternal and newborn health
	Home-based records

Smith H, *et al. BMJ Glob Health* 2024; 8:e014033. doi: 10.1136/bmjgh-2023-014033

Table S6. Key international legal documents related to postnatal care (primary international law documents) not included in the mapping (n=8)

Documents (n=8) (in chronological order)	Abbreviation	Year of adoption	Year entered into force (for treaties)	Treaty monitoring body (TMB)*	Abbreviation
**Universal Declaration of Human Rights** (Article 25)	UDHR	1948	—	United Nations	UN
**C102 – Social Security Convention** (Articles 10 and 49)	—	1952	—	International Labour Organization	ILO
**International Covenant on Civil and Political Rights** (Article 24)	ICCPR	1966	1976	Human Rights Committee	HRC
**International Covenant on Economic, Social and Cultural Rights** (Articles 9, 10, 12 and 15)	ICESCR	1966	1976	Committee on Economic, Social and Cultural Rights	CESCR
**Convention on the Elimination of All Forms of Discrimination against Women** (Articles 2, 5, 11, 12, 13 and 16)	CEDAW	1979	1981	Committee on the Elimination of Discrimination against Women	CEDAW
**C156 – Workers with Family Responsibilities Convention** (Articles 4 and 5)	—	1981	—	International Labour Organization	ILO
**Convention on the Rights of the Child** (Articles 7, 9 and 24)	CRC	1989	1990	Committee on the Rights of the Child	CRC

**C183 – Maternity Protection Convention**<br>(Articles 4, 6, and 10)	—	2000	—	International Labour Organization	ILO

Table S7: Additional documents (broader statements on human rights or the application of human rights approaches) not included in mapping exercise (n=8)

Document	Relevance
**Special Rapporteur on the right of everyone to the enjoyment of the highest attainable standard of physical and mental health, Report. UN Doc. E/CN.4/2004/49; 2004**	Considers sexual and reproductive health through the prism of the right to health. “The Special Rapporteur takes the view that the rights to sexual and reproductive health have an indispensable role to play in the struggle against intolerance, gender inequality, HIV/AIDS and poverty, and he recommends that increased attention be devoted to a proper understanding of reproductive health, reproductive rights, sexual health and sexual rights.”
**World Health Organization, Office of the United Nations High Commissioner for Human Rights. A human rights-based approach to health. Geneva: World Health Organization, 2008**	Describes the broader application of human rights-based approaches to health.
**Special Rapporteur on the right of everyone to the enjoyment of the highest attainable standard of physical and mental health, Interim Report to the General Assembly. UN Doc. A/66/254; 2011**	“In the present report, the Special Rapporteur on the right of everyone to the enjoyment of the highest attainable standard of physical and mental health considers the interaction between criminal laws and other legal restrictions relating to sexual and reproductive health and the right to health…”
**Global Strategy for Women’s, Children’s and Adolescents’ Health (2016–2030). New York: Every Woman Every Child, 2015**	Outlines a roadmap to achieve right to the highest attainable standard of health for all women, children and adolescents.
**Report of the Special Rapporteur on the Right of everyone to the enjoyment of the highest attainable standard of physical and mental health. A/70/213; 2015**	Presents a case for increased investments in individual and societal health from all relevant actors, including in the post 2015 agenda.
**World Health Organization. Leading the realization of human rights to health and through health: report of the high-Level working group on the health and human rights of women, children and adolescents. Geneva: World Health Organization, 2017**	Presents a roadmap for implementation of the human rights-related measures required by the Global Strategy for Women’s, Children’s and Adolescents’ Health (2016-2030).
**ODI/UNICEF. Universal child benefits: policy issues and options. London: Overseas Development Institute and New York: UNICEF, 2020**	Report critically reviews the case for universal child benefits (UCBs), including the (potential) role of UCBs as a policy instrument in the pursuit of child poverty reduction and universal social protection.
**A/76/172: Report of the Special Rapporteur on the right of everyone to the enjoyment of the highest attainable standard of physical and mental health, Tlaleng Mofokeng - Sexual and reproductive health rights: challenges and opportunities during the COVID-19 pandemic, 2021**	Describes how the COVID-19 pandemic has impacted on the realisation of the sexual and reproductive health rights of women, adolescents, girls and all persons capable of getting pregnant.

ODI: Overseas Development Institute; UNICEF: United Nations Childrens Fund

Smith H, *et al. BMJ Glob Health* 2024; 8:e014033. doi: 10.1136/bmjgh-2023-014033

Table S8: Summary of content extracted from documents used in mapping (n=29)

Document	Article/section	Examples of extracted content
**INTERNATIONAL LEGAL AND POLITICAL COMMITMENTS**		
**HRC Resolution 22/7: Birth registration and the right of everyone to recognition everywhere as a person before the law** ***2013***	Various paragraphs	*…Recalling the obligation of States to* ***register all children immediately after birth****, as provided for in the International Covenant on Civil and Political Rights, the Convention on the Rights of the Child, the International Convention on the Rights of All Migrant Workers and Members of Their Families and other relevant international instruments to which they are party,*<br><br>*Recognizing the* ***importance of a human rights-based approach to birth registration****, based on international human rights obligations and commitments operationally directed to promoting and protecting human rights,*<br><br>*Recognizing also the* ***importance of birth registration, including late birth registration, for the development of vital statistics and the effective implementation of programmes and policies*** *intended to achieve internationally agreed development goals, including the Millennium Development Goals,*
**HRC Resolution 28/13: Birth registration and the right of everyone to recognition everywhere as a person before the law** ***2015***		As above
**HRC Resolution 33/11: Preventable mortality and morbidity of children under 5 years of age as a human rights concern.** ***2016***	Page 2<br><br>Para 3.	*Reaffirming that States* ***should take all appropriate measures to ensure the right of the child to the enjoyment of the highest attainable standard of physical and mental health without discrimination of any kind*** *and, in doing so, be guided by the best interests of the child, ensuring the meaningful participation of children, consistent with their evolving capacities, in all matters and decisions affecting their lives, bearing in mind the rights, duties and responsibilities of parents or caregivers in relation to preventing mortality and morbidity of children under 5 years of age, and take steps to ensure the allocation of available resources to the maximum extent possible to achieve the full realization of the right of the child to the highest attainable standard of health, including by strengthening international cooperation in this field*<br><br>*3. Calls upon States to adopt a human rights-based approach to reducing and eliminating preventable mortality and morbidity of children under 5 years of age****, including in scaling up efforts to achieve the integrated management of quality maternal, newborn and child health care and services****, particularly at the community and family levels, and to take action to address the main causes of preventable mortality and morbidity of children under 5 years of age;*
**HRC Resolution 33/18: Preventable maternal mortality and morbidity and human rights.** ***2016***	Page 2	*Recognizing that* ***sexual and reproductive health and rights are integral to the progressive realization of the right of everyone to the enjoyment of the highest attainable standard of physical and mental health****, and that comprehensive sexual and reproductive health care and services contain the interrelated and essential elements of availability, accessibility, affordability, acceptability and quality, on the basis of non-discrimination and formal and substantive equality, while including the need to address intersectional and multiple forms of discrimination,*
**HRC Resolution adopted by the Human Rights Council on 24 March 2017. 34/15: Birth registration and the right of everyone to recognition everywhere as a person before the law** ***2017***	Various paragraphs	*…Recalling the* ***obligation of States to register all children, without discrimination of any kind, immediately after birth, which is an important element of the protection and realization of all human rights,*** *as provided for in the International Covenant on Civil and Political Rights, the Convention on the Rights of the Child, the International Convention on the Protection of the Rights of All Migrant Workers and Members of Their Families and other relevant international instruments to which they are party,*<br><br>*Recognizing that* ***birth registration and the right to recognition everywhere as a person before the law is closely linked to the realization of all other human rights****, and therefore underlining the importance of a human rights-*

Document	Article/section	Examples of extracted content
		based approach to birth registration, based on international human rights obligations and commitments operationally directed to promoting and protecting human rights, …<br><br>*Recognizing that the* ***full implementation of this target will have both a direct and an indirect impact on the achievement of other targets and goals, inter alia social protection, protection in emergencies, access to financial and economic resources, the elimination of all forms of discrimination and violence against women and children everywhere****, and access to quality education,*<br><br>*Recognizing that* ***free birth registration and free or low-fee late birth registration are part of a comprehensive civil registration system that facilitates the development of vital statistics and the effective planning and implementation of programmes and policies*** *intended to promote better governance and to achieve internationally agreed development goals,*
HRC Resolution 41/14: Equal pay. ***2019***	Para 2. (d) and (f)	*2 (d) To design, implement and promote family-responsive legislation, policies and services, such as* ***shared parental and other leave schemes, increased flexibility in healthy and safe working arrangements for women and men, without reductions in labour and social protection, support for breastfeeding mothers, development of infrastructure and technology, and the provision of services, including universal affordable, accessible and quality care facilities for children and other dependants, which create an enabling environment for women's labour market participation and their economic independence;***<br><br>*2 (f) To take all appropriate* ***measures to address the wage disparity and reduction experienced by many women when they have children****, including by promoting parental and paternity leave and men's use of such leave through, inter alia, dedicated, non-transferable paid leave for fathers, and by ensuring that such leave is connected to the availability of affordable, accessible, inclusive and quality childcare services and facilities, including early childhood services and after-school services for children and adolescents, and to ensure a seamless transition of parents back into the labour market;*
HRC Resolution 41/17: Preventing and responding to violence against women and girls in the world of work. ***2019***	Page 3	*Acknowledging that some forms of violence in the world of work affect women specifically, such as harassment, discrimination and bullying by co-workers, subordinates or superiors* ***in the context of pregnancy, breastfeeding and maternity leave***
HRC Special Rapporteur A/74/137: A human-rights based approach to mistreatment and violence against women in reproductive health services with a focus on childbirth and obstetric violence. ***2019***	Section A; para 5.<br><br>Section C; para 23.<br><br>Section E; para 54.	*5. …the World Health Organization (WHO) reacted to the growing concerns of women during childbirth by issuing a statement in 2015* ***condemning "outright*** *physical abuse, profound humiliation and verbal abuse, coercive or unconsented medical procedures (including sterilization), lack of confidentiality, failure to get fully informed consent, refusal to give pain medication, gross violations of privacy, refusal of admission to health facilities, neglecting women during childbirth to suffer life-threatening, avoidable complications, and* ***detention of women and their newborns in facilities after childbirth due to an inability to pay****."*<br><br>*23. The* ***post-childbirth detention of women and their newborns in health-care facilities because of their inability to pay hospital fees is another example of a human rights violation****. This practice has been reported in a number of countries in Asia, sub - Saharan Africa, Latin America and the Middle East. In Kenya, detained women and their infants have been made to sleep on the floor, denied adequate food and watched over by guards. There are reports of women and their children spending weeks, and even years, in such conditions. In its concluding observations, the Committee against Torture has condemned the "practice of post-delivery detention of women unable to pay their medical bills" in Kenya.*

Document	Article/section	Examples of extracted content
		54. …Article 12 of the Convention recommends that ***States guarantee women "appropriate services in connection with pregnancy, confinement and the post-natal period, granting free services where necessary"***
Innocenti Declaration on the protection, promotion and support of breastfeeding. ***1990***	Whole document	
WHA 27.43: Infant nutrition and breastfeeding. ***1974***	Para 2, 4.	*2. CALLS THE ATTENTION of countries to the* ***necessity of taking adequate social measures for mothers working away from their homes during the lactation period, such as arranging special work timetables*** *so that they can breast-feed their children*<br><br>*4. URGES the Director-General to intensify activities relevant to the promotion of breast-feeding, to* ***bring those matters to the notice of the medical profession and health administrators and to emphasize the need for health personnel, mothers and the general public to be educated*** *accordingly*
WHA 55.25: Infant and young child nutrition. ***2002***	Para 2 (2)	*2. URGES Member States, as a matter of urgency:*<br><br>*(1) to adopt and implement the global strategy, taking into account national circumstances, while respecting positive local traditions and values, as part of their overall nutrition and child health policies and programmes, in order to ensure optimal feeding for all infants and young children, and to reduce the risks associated with obesity and other forms of malnutrition;*<br><br>*(2)* ***to strengthen existing, or establish new, structures for implementing the global strategy through the health and other concerned sectors, for monitoring and evaluating its effectiveness, and for guiding resource investment and management to improve infant and young-child feeding;***
WHA 69: Maternal, infant and young child nutrition: guidance on ending the inappropriate promotion of foods for infants and young children. ***2016***	Para 11, 16.	*11. Recommendation 2.* ***Products that function as breast-milk substitutes should not be promoted****. A breast-milk substitute should be understood to include any milks (or products that could be used to replace milk, such as fortified soy milk), in either liquid or powdered form, that are specifically marketed for feeding infants and young children up to the age of 3 years (including follow-up formula and growing-up milks). It should be clear that the implementation of the International Code of Marketing of Breast-milk Substitutes and subsequent relevant Health Assembly resolutions covers all these products.*<br><br>*16. Recommendation 6.* ***Companies that market foods for infants and young children should not create conflicts of interest in health facilities or throughout health system****s. Health workers, health systems, health professional associations and nongovernmental organizations should likewise avoid such conflicts of interest…*
**INTERPRETATIONS OF LEGAL AND POLITICAL COMMITMENTS**		
ICESCR GC No. 14: The right to the highest attainable standard of health (Art. 12). ***E/C.12/2000/4; 2000***	Para. 14, 21, 22	*14." The provision for the reduction of the stillbirth rate and of infant mortality and for the healthy development of the child" (art. 12.2 (a))10 may be understood as requiring measures to improve child and maternal health, sexual and reproductive health services, including* ***access to family planning, pre- and post-natal care****, emergency obstetric services and access to information, as well as to resources necessary to act on that information.*<br><br>*Women and the right to health*<br><br>*21. To eliminate discrimination against women, there is a need to develop and implement a comprehensive national strategy for promoting* ***women's right to health throughout their life span****. Such a strategy should include interventions aimed at the prevention and treatment of diseases affecting women, as well as policies to provide access to* ***a full range of high quality and affordable health care, including sexual and reproductive services****. A*

Document	Article/section	Examples of extracted content
		major goal should be reducing women's health risks, particularly lowering rates of maternal mortality and protecting women from domestic violence. The realization of women's right to health requires the removal of all barriers interfering with access to health services, education and information, including in the area of sexual and reproductive health. It is also important to undertake preventive, promotive and remedial action to shield women from the impact of harmful traditional cultural practices and norms that deny them their full reproductive rights.<br><br>*Children and adolescents*<br><br>*22. Article 12.2 (a) outlines the need to take measures to reduce infant mortality and promote the healthy development of infants and children. Subsequent international human rights instruments recognize that* ***children and adolescents have the right to the enjoyment of the highest standard of health and access to facilities*** *for the treatment of illness. The Convention on the Rights of the Child directs States to ensure* ***access to essential health services for the child and his or her family, including pre- and post-natal care for mothers****. The Convention links these goals with ensuring access to child-friendly information about preventive and health-promoting behaviour and support to families and communities in implementing these practices. Implementation of the principle of non-discrimination requires that girls, as well as boys, have equal access to adequate nutrition, safe environments, and physical as well as mental health services. There is a need to adopt effective and appropriate measures to abolish harmful traditional practices affecting the health of children, particularly girls, including early marriage, female genital mutilation, preferential feeding and care of male children. Children with disabilities should be given the opportunity to enjoy a fulfilling and decent life and to participate within their community.*
ICESCR GC No. 19: The right to social security (Art. 9). ***E/C.12/GC/19; 2008***	Para. 1 and 2<br><br>Section A; subsection 2; (f) and (g)	*(f) Family and child support*<br><br>*18.* ***Benefits for families are crucial for realizing the rights of children*** *and adult dependents to protection under articles 9 and 10 of the Covenant. In providing the benefits, the State party should take into account the resources and circumstances of the child and persons having responsibility for the maintenance of the child or adult dependent, as well as any other consideration relevant to an application for benefits made by or on behalf of the child or adult dependent. Family and child benefits, including cash benefits and social services, should be provided to families, without discrimination on prohibited grounds, and would ordinarily cover food, clothing, housing, water and sanitation, or other rights as appropriate.*<br><br>*(g) Maternity*<br><br>*19. Article 10 of the Covenant expressly provides that* ***"working mothers should be accorded paid leave or leave with adequate social security benefits". Paid maternity leave should be granted to all women****, including those involved in atypical work, and benefits should be provided for an adequate period.* ***Appropriate medical benefits should be provided for women and children, including perinatal, childbirth and postnatal care*** *and care in hospital where necessary.*
ICESCR GC No. 22 on the right to sexual and reproductive health (Art. 12 of the ICESCR). ***E/C.12/GC/22; 2016***	Para. 9, 11, 25	*9. …The right to sexual and reproductive health, combined with the right to work (article 6) and just and favourable working conditions (article 7), as well as the right to non-discrimination and equality between men and women,* ***also requires States to ensure employment with maternity protection and parental leave for workers,*** *including workers in vulnerable situations, such as migrant workers or women with disabilities, as well as protection from sexual harassment in the workplace and prohibition of discrimination based on pregnancy, childbirth, parenthood, sexual orientation, gender identity or intersex status.*

Document	Article/section	Examples of extracted content
		11.The ***right to sexual and reproductive health is an integral part of the right of everyone to the highest attainable physical and mental health****. Following the elaboration in the Committee's general comment No. 14, comprehensive sexual and reproductive health care contains the four interrelated and essential elements described below.*<br><br>*25.Due to women's reproductive capacities, the* ***realization of the right of women to sexual and reproductive health is essential to the realization of the full range of their human rights****. The right of women to sexual and reproductive health is indispensable to their autonomy and their right to make meaningful decisions about their lives and health. Gender equality requires that the health needs of women, different from those of men, be taken into account and appropriate services provided for women in accordance with their life cycles.*
CEDAW GC No. 24: Article 12 of the Convention (Women and Health). ***A/54/38/Rev.1, chap. I; 1999***	Article 12; para 8<br><br>Article 12 (1); para 12, 23, 26	*Article 12 reads as follows:*<br><br>*"1. States parties shall take all appropriate measures to eliminate discrimination against women in the field of health care in order to ensure, on a basis of equality of men and women, access to health-care services, including those related to family planning.*<br><br>*"2. Notwithstanding the provisions of paragraph 1 of this article,* ***States parties shall ensure to women appropriate services in connection with pregnancy, confinement and the post-natal period, granting free services where necessary****, as well as adequate nutrition during pregnancy and lactation."…*<br><br>Other relevant content:<br><br>*12. States parties should report on their understanding of how policies and measures on health care address the health rights of women from the perspective of women's needs and interests and how it addresses distinctive features and factors that differ for women in comparison to men, such as:*<br><br>*…(c)* ***Psychosocial factors that vary between women and men include depression in general and post-partum depression in particular*** *as well as other psychological conditions, such as those that lead to eating disorders such as anorexia and bulimia;*<br><br>*23. In their reports,* ***States parties should state what measures they have taken to ensure timely access to the range of services that are related to family planning****, in particular, and to sexual and reproductive health in general. Particular attention should be paid to the health education of adolescents, including information and counselling on all methods of family planning.**<br><br>*26.* ***Reports should also include what measures States parties have taken to ensure women appropriate services in connection with pregnancy, confinement and the post-natal period.*** *Information on the rates at which these measures have reduced maternal mortality and morbidity in their countries, in general, and in vulnerable groups, regions and communities, in particular, should also be included.*
ICESCR GC No. 25: Science and economic, social and cultural rights (Article 15(1)(b), (2), (3) and (4) of the ICESCR). ***E/C.12/GC/25; 2020***	Section D; para 66	*66. Inadequate diets have become a major contributing factor to the increase of non-communicable diseases in all regions. Given the proven long-term effects of adequate nutrition during pregnancy and before a child's second birthday,* ***States should do more to regulate the marketing of breast milk substitutes, to disseminate information about the benefits of adequate feeding practices, and to create an enabling environment for breastfeeding.*** *They should also redirect investments in agricultural development away from the exclusive focus on boosting the production of cereal crops – rice, wheat and maize – towards support for healthy diets, including adequate measures to reduce the excessive intake of sugar. Cereal crops are mainly a source of carbohydrates and contain relatively few proteins and other nutrients essential for adequate diets.*

Document	Article/section	Examples of extracted content
CRC GC No. 15 on the right of the child to the enjoyment of the highest attainable standard of health (Art. 24). ***CRC/C/GC/15; 2013***	Para. 2<br><br>Section D; para 18<br><br>Article 24, para 2 (d); subsections 51-57.<br><br>Article 24, para 2 (f); subsections 62-70	*Various paragraphs, including:*<br><br>*2. The Committee interprets children's right to health as defined in article 24 as an* ***inclusive right, extending not only to timely and appropriate prevention, health promotion, curative, rehabilitative and palliative services, but also to a right to grow and develop to their full potential and live in conditions that enable them to attain the highest standard of health through the implementation of programmes that address the underlying determinants of health****. A holistic approach to health places the realization of children's right to health within the* ***broader framework of international human rights obligations.***<br><br>*18. Among the* ***key determinants of children's health, nutrition and development are the realization of the mother's right to health and the role of parents and other caregivers****. A significant number of infant deaths occur during the neonatal period, related to the poor health of the mother prior to, and during, the pregnancy and the immediate post-partum period, and to suboptimal breastfeeding practices. The health and health-related behaviours of parents and other significant adults have a major impact on children's health.*<br><br>*2 (d). "To ensure appropriate pre-natal and post-natal care for mothers" …*<br><br>*2 (f) "To develop preventive health care, guidance for parents and family planning education and services"…*
CRC GC No. 7. Implementing child rights in early childhood. ***CRC/C/GC/7/Rev.1; 2005***	Section III; para 10<br><br>Section V; para 25, 27 (b), 28	*10.* ***Right to life, survival and development****. Article 6 refers to the child's inherent right to life and States parties' obligation to ensure, to the maximum extent possible, the survival and development of the child. States parties are urged to take all possible measures to improve perinatal care for mothers and babies, reduce infant and child mortality, and create conditions that promote the well-being of all young children during this critical phase of their lives…*<br><br>*25.* ***Birth registration****. Comprehensive services for early childhood begin at birth. The Committee notes that provision for registration of all children at birth is still a major challenge for many countries and regions. This can impact negatively on a child's sense of personal identity and children may be denied entitlements to basic health, education and social welfare. As a first step in ensuring the rights to survival, development and access to quality services for all children (art. 6), the Committee recommends that States parties take all necessary measures to ensure that all children are registered at birth… The Committee also reminds States parties of the importance of facilitating late registration of birth, and ensuring that children who have not been registered have equal access to health care, protection, education and other social services.*<br><br>*27 (b). States parties have a responsibility to implement children's right to health by encouraging education in child health and development, including about the advantages of breastfeeding, nutrition, hygiene and sanitation. Priority should also be given to the* ***provision of appropriate prenatal and post-natal health care for mothers and infants in order to foster healthy family-child relationships, especially between a child and his or her mother (or other primary caregiver****) (art. 24.2). Young children are themselves able to contribute to ensuring their personal health and encouraging healthy lifestyles among their peers, for example through participation in appropriate, child-centred health education programmes;*<br><br>*28.* ***Early childhood education****. The Convention recognizes the right of the child to education, and primary education should be made compulsory and available free to all (art. 28). The Committee recognizes with appreciation that some States parties are planning to make one year of preschool education available and free of cost for all children. The Committee interprets the right to education during early childhood as beginning at birth and closely linked to young children's right to maximum development (art. 6.2)…*

Smith H, *et al. BMJ Glob Health* 2024; 8:e014033. doi: 10.1136/bmjgh-2023-014033

Document	Article/section	Examples of extracted content
ILO: Income Security Recommendation R067. ***1944***	Annex Part 1 Social insurance. Para. 10	*Maternity*<br><br>*10. The contingency for which* ***maternity benefit should be paid is loss of earnings due to abstention from work during prescribed periods before and after childbirth.***<br><br>*(1) A woman should have the right to leave her work if she produces a medical certificate stating that her confinement will probably take place within six weeks, and no woman should be permitted to work during the six weeks following her confinement.*<br><br>*(2) During these periods maternity benefit should be payable.*<br><br>*(3) Absence from work for longer periods or on other occasions may be desirable on medical grounds, having regard to the physical condition of the beneficiary and the exigencies of her work; during any such periods sickness benefits should be payable.*<br><br>*(4) The payment of maternity benefit may be made conditional on the utilisation by the beneficiary of health services provided for her and her child.*
ILO: Workers with Family Responsibilities Recommendation R165. ***1981***	Paras 22 and 28	*22. (1) Either parent should have the possibility, within a period immediately following maternity leave, of obtaining leave of absence (parental leave), without relinquishing employment and with rights resulting from employment being safeguarded.*<br><br>*(2) The length of the period following maternity leave and the duration and conditions of the leave of absence referred to in subparagraph (1) of this Paragraph should be determined in each country by one of the means referred to in Paragraph 3 of this Recommendation.*<br><br>*(3) The leave of absence referred to in subparagraph (1) of this Paragraph may be introduced gradually.*<br><br>*28. During the leave of absence referred to in Paragraphs 22 and 23, the workers concerned may, in conformity with national conditions and practice, and by one of the means referred to in Paragraph 3 of this Recommendation, be protected by social security.*
ILO: Maternity Protection Recommendation R191. ***2000***	Various paragraphs	***Maternity leave***<br><br>*1. (1) Members should endeavour to extend the period of maternity leave referred to in Article 4 of the Convention to at least 18 weeks. (2) Provision should be made for an extension of the maternity leave in the event of multiple births. (3) To the extent possible, measures should be taken to ensure that the woman is entitled to choose freely the time at which she takes any non-compulsory portion of her maternity leave, before or after childbirth.*<br><br>***Benefits***<br><br>*2. Where practicable, and after consultation with the representative organizations of employers and workers, the cash benefits to which a woman is entitled during leave referred to in Articles 4 and 5 of the Convention should be raised to the full amount of the woman's previous earnings or of such of those earnings as are taken into account for the purpose of computing benefits.*<br><br>*3. To the extent possible, the medical benefits provided for in Article 6, paragraph 7, of the Convention should include: (a) care given in a doctor's office, at home or in a hospital or other medical establishment by a general practitioner or a specialist; (b) maternity care given by a qualified midwife or by another maternity service at home or in a hospital or other medical establishment; (c) maintenance in a hospital or other medical establishment; (d) any*

Document	Article/section	Examples of extracted content
		necessary pharmaceutical and medical supplies, examinations and tests prescribed by a medical practitioner or other qualified person; and (e) dental and surgical care.<br>***Financing of benefits***<br>*4. Any contribution due under compulsory social insurance providing maternity benefits and any tax based upon payrolls which is raised for the purpose of providing such benefits, whether paid by both the employer and the employees or by the employer, should be paid in respect of the total number of men and women employed, without distinction of sex.*<br>***Employment protection and non-discrimination***<br>*5. A woman should be entitled to return to her former position or an equivalent position paid at the same rate at the end of her leave referred to in Article 5 of the Convention. The period of leave referred to in Articles 4 and 5 of the Convention should be considered as a period of service for the determination of her rights.*<br>***Health protection***<br>*6. (1) Members should take measures to ensure assessment of any workplace risks related to the safety and health of the pregnant or nursing woman and her child. The results of the assessment should be made available to the woman concerned.*<br>*(2) In any of the situations referred to in Article 3 of the Convention or where a significant risk has been identified under subparagraph (1) above, measures should be taken to provide, on the basis of a medical certificate as appropriate, an alternative to such work in the form of (a) elimination of risk; (b) an adaptation of her conditions of work ;(c) a transfer to another post, without loss of pay, when such an adaptation is not feasible; or (d) paid leave, in accordance with national laws, regulations or practice, when such a transfer is not feasible.*<br>*(3) Measures referred to in subparagraph (2) should in particular be taken in respect of: (a) arduous work involving the manual lifting, carrying, pushing or pulling of loads; (b) work involving exposure to biological, chemical or physical agents which represent a reproductive health hazard; (c) work requiring special equilibrium; (d) work involving physical strain due to prolonged periods of sitting or standing, to extreme temperatures, or to vibration.*<br>*(4) A pregnant or nursing woman should not be obliged to do night work if a medical certificate declares such work to be incompatible with her pregnancy or nursing.*<br>*(5) The woman should retain the right to return to her job or an equivalent job as soon as it is safe for her to do so*<br>*(6) A woman should be allowed to leave her workplace, if necessary, after notifying her employer, for the purpose of undergoing medical examinations relating to her pregnancy.*<br>***Breastfeeding mothers***<br>*7. On production of a medical certificate or other appropriate certification as determined by national law and practice, the frequency and length of nursing breaks should be adapted to particular needs.*<br>*8. Where practicable and with the agreement of the employer and the woman concerned, it should be possible to combine the time allotted for daily nursing breaks to allow a reduction of hours of work at the beginning or at the end of the working day.*

Document	Article/section	Examples of extracted content
		9. Where practicable, provision should be made for the establishment of facilities for nursing under adequate hygienic conditions at or near the workplace.<br><br>***Related types of leave***<br><br>*10. (1) In the case of the death of the mother before the expiry of postnatal leave, the employed father of the child should be entitled to take leave of a duration equal to the unexpired portion of the postnatal maternity leave.*<br><br>*(2) In the case of sickness or hospitalization of the mother after childbirth and before the expiry of postnatal leave, and where the mother cannot look after the child, the employed father of the child should be entitled to leave of a duration equal to the unexpired portion of the postnatal maternity leave, in accordance with national law and practice, to look after the child.*<br><br>*(3) The employed mother or the employed father of the child should be entitled to parental leave during a period following the expiry of maternity leave.*<br><br>*(4) The period during which parental leave might be granted, the length of the leave and other modalities, including the payment of parental benefits and the use and distribution of parental leave between the employed parents, should be determined by national laws or regulations or in any manner consistent with national practice.*<br><br>*(5) Where national law and practice provide for adoption, adoptive parents should have access to the system of protection offered by the Convention, especially regarding leave, benefits and employment protection.*
**ILO: Social Protection Floors Recommendation R202.** ***2012***	Part 2; national social protection floors; para 5.	*5. The social protection floors referred to in Paragraph 4 should comprise at least the following basic social security guarantees:*<br><br>*(a) access to a* ***nationally defined set of goods and services, constituting essential health care, including maternity care****, that meets the criteria of availability, accessibility, acceptability and quality;*<br><br>*(b)* ***basic income security for children****, at least at a nationally defined minimum level, providing access to nutrition, education, care and any other necessary goods and services;*<br><br>*(c)* ***basic income security****, at least at a nationally defined minimum level, for persons in active age who are unable to earn sufficient income, in particular in cases of sickness, unemployment,* ***maternity*** *and disability; and*<br><br>*(d) basic income security, at least at a nationally defined minimum level, for older persons.*
**Joint WHO/UNICEF meeting on Infant and Young Child Feeding: statement, recommendations, list of participants.** ***1981***	Page 12, 13,	*After delivery*<br><br>*All* ***postnatal health care should be oriented towards ensuring the maintenance of breastfeeding for as long as possible****. All babies should receive colostrum. For optimal breastfeeding, the use of supplementary bottle-feeding – water and formula – should be avoided. A healthy well-nourished mother who is fully breastfeeding her infant should not need to introduce any complements until after the first 4-6 months of life, according to the needs of the infant.*<br><br>*Mothers' nutritional status should be reviewed and, whenever possible, steps taken to ensure that the mother has access to adequate food intake.*<br><br>*The contraceptive effect of breastfeeding should be well recognized, although additional family planning methods should be promoted to ensure birth spacing. Preference should be given to contraceptive methods which do not interfere with the normal process of lactation.*

Document	Article/section	Examples of extracted content
		*All attempts should be made to ensure that **in cases where infants need to be hospitalized facilities are provided so that the mother can be with the infant and continue breastfeeding or that the baby can continue to receive breastmilk**. Where it is not possible for the biological mother to breastfeed, the first alternative, if available, should be the use of human breastmilk from other sources. Human milk banks should be made available in appropriate situations.*<br><br>*Support through health services*<br><br>*Health service staff must **play a critical role in the initiation,** establishment and maintenance of breastfeeding and **should ensure that the mother has a source of sustained support for as long as breastfeeding continues, and thus health workers should he well informed and provide consistent information**.*<br><br>*Employed mothers*<br><br>***Paid maternity leave of not less than three months postnatal, job security and economic support should be provided to all mothers whenever possible**, and wherever possible, and the responsibility for economic support during maternity leave should be carried by government, the industry in which the woman is working, and other relevant national and international institutions. Creches, **paid breastfeeding breaks and other facilities should be provided** wherever appropriate, in industry, and in other relevant institutions, or close to the place of work to permit mothers to continue breastfeeding and have close contact with their babies. Financing of creches and other mechanisms that allow for this continued contact of breastfeeding should be carried by government and/or the industry in which the mother is working.*
Joint statement by the UN Special Rapporteurs: Right to Food, Right to Health, Working Group on Discrimination against Women in law and in practice, and RC in support of increased efforts to promote, support and protect breast-feeding. ***2016***	Whole document	
The prevention and elimination of disrespect and abuse during facility-based childbirth. ***WHO, 2014***	Page 1	*Reports of disrespectful and abusive treatment during childbirth in facilities have included outright physical abuse, profound humiliation and verbal abuse, coercive or unconsented medical procedures (including sterilization), lack of confidentiality, failure to get fully informed consent, refusal to give pain medication, gross violations of privacy, refusal of admission to health facilities, neglecting women during childbirth to suffer life-threatening, avoidable complications, and **detention of women and their newborns in facilities after childbirth due to an inability to pay**.*
Ensuring human rights in the provision of contraceptive information and services. ***WHO, 2014***	Whole document	
Global strategy for infant and young child feeding. ***WHO, 2003***	Para 27 and 34	*27. Mothers should have access to **skilled support to help them initiate and sustain appropriate feeding practices**, and to prevent difficulties and overcome them when they occur. Knowledgeable health workers are well placed to provide this support, which should be a routine part not only of regular prenatal, delivery and **postnatal care** but also of services provided for the well baby and sick child. Community-based networks offering mother-to-mother support, and trained **breastfeeding counsellors working within, or closely with, the health care system**, also have an important role to play in this regard. Where fathers are concerned, research shows that breastfeeding is enhanced by the support and companionship they provide as family providers and caregivers.*

Smith H, *et al. BMJ Glob Health* 2024; 8:e014033. doi: 10.1136/bmjgh-2023-014033

Document	Article/section	Examples of extracted content
		34. A comprehensive national policy, based on a thorough needs assessment, should foster an ***environment that protects, promotes and supports appropriate infant and young child feeding practices****. An effective feeding policy consistent with efforts to promote overall household food security requires the following critical interventions:*<br>*For protection…*<br>*For promotion…*<br>*For support through the health care system…*<br>*For support in the community…*<br>*For support for feeding infants and young children in exceptionally difficult circumstances…*
**Ending hospital detention for non-payment of bills: legal and health financing policy options.** ***WHO, 2020***	Whole document	

CEDAW: Convention on the Elimination of All Forms of Discrimination against Women; CRC: Convention on the Rights of the Child; GC: general comment; HRC: UN Human Rights Council; ICESCR: Convention on Economic, Social and Cultural Rights; ILO: International Labour Organization; UN: United Nations; UNICEF: United Nations Children's Fund; WHA: World Health Assembly; WHO: World Health Organization

Smith H, *et al. BMJ Glob Health* 2024; 8:e014033. doi: 10.1136/bmjgh-2023-014033
